# Detection of Free-Living Amoebae Using Amoebal Enrichment in a Wastewater Treatment Plant of Gauteng Province, South Africa

**DOI:** 10.1155/2014/575297

**Published:** 2014-11-04

**Authors:** P. Muchesa, O. Mwamba, T. G. Barnard, C. Bartie

**Affiliations:** ^1^Water and Health Research Centre, University of Johannesburg, P.O. Box 17011, Doornfontein 2028, South Africa; ^2^National Institute for Occupational Health, P.O. Box 4788, Johannesburg 2000, South Africa

## Abstract

Free-living amoebae pose a potential health risk in water systems as they may be pathogenic and harbor potential pathogenic bacteria known as amoebae resistant bacteria. Free-living amoebae were observed in 150 (87.2%) of the environmental water samples. In particular, *Acanthamoeba* sp. was identified in 22 (12.8%) using amoebal enrichment and confirmed by molecular analysis. FLA were isolated in all 8 stages of the wastewater treatment plant using the amoebal enrichment technique. A total of 16 (9.3%) samples were positive for FLA from influent, 20 (11.6%) from bioreactor feed, 16 (9.3%) from anaerobic zone, 16 (9.3%) from anoxic zone, 32 (18.6%) from aerators, 16 (9.3%) from bioreactor effluent, 11 (6.4%) from bioreactor final effluent, and 45 (26.2%) from maturation pond. This study provides baseline information on the occurrence of amoebae in wastewater treatment plant. This has health implications on receiving water bodies as some FLA are pathogenic and are also involved in the transmission and dissemination of pathogenic bacteria.

## 1. Introduction

Free-living amoebae (FLA) are unicellular protozoa that exist in high numbers in aquatic environments where they play a useful role as predators of bacteria, algae, viruses, and fungi [1]. They have been isolated from process water systems such as cooling towers, hospital water networks, and drinking and wastewater water plants [2–4].* Naegleria fowleri, Balamuthia mandrillaris, *and* Acanthamoeba* are some of the FLA species known to be pathogenic to humans [5–7].* Acanthamoeba *species are the causative agent of amoebic keratitis (AK) and granulomatous amoebic encephalitis (GAE) while* Naegleria fowleri *and* Balamuthia mandrillaris *have been associated with amoebic meningoencephalitis (PAM) and GAE, respectively [6]. This study focused on detection of these pathogenic FLA, specificallythe* Acanthamoeba *sp., in a wastewater treatment plant using an optimized amoebal enrichment technique.

Most FLA have two developmental stages (some FLA also have a flagellate intermediate form): an active trophozoite stage and a dormant cyst stage. Trophozoites actively feed through phagocytosis and pinocytosis on microorganisms and small organic particles in the environment [8, 9]. The cyst stage occurs when environmental conditions are unfavourable, for example, in extremes of temperature, osmotic pressure, and pH, or when nutrient levels are depleted. FLA can survive in the cyst stage for extended periods of time, only to become active trophozoites when environmental conditions become favourable again [6, 10]. These amoebal cysts contain cellulose which forms a physical protective barrier making them resistant to a wide variety of water treatment regimes. Some studies have reported survival of amoebal cysts after clarification, rapid filtration, and ultrafiltration processes, as well as after biocide treatment. Biocides such as chlorine, chlorine dioxide, monochloramine, ozone, copper-silver nitrate, and ultraviolet light have shown limited success against a variety of amoebal cysts in water treatment systems [11–13]. This has huge implications in water treatment systems for drinking water and sewage treatment in South Africa which relies heavily on chlorine as biocidal for water treatment.

Free-living amoebae can also act as reservoirs of pathogenic bacteria such as methicillin-resistant* Staphylococcus aureus*,* Vibrio cholerae*,* Legionella *species including* Legionella pneumophila,* and environmental* Mycobacterium* species as reviewed by Goñi et al. [14]. These “amoebae resistant bacteria” (ARB) are able to infect and resist the digestive process of FLA, survive, multiply, and exit FLA enabling them to spread and colonize aquatic water systems [15–17]. The list of confirmed ARB currently stands at 102 species and continues to grow [18]. ARB use their amoebal hosts for nutrition and protection (when amoebae form cysts) during harsh environmental conditions such as in the presence of biocides like chlorine used in water treatment. Some genera, particularly,* L. pneumophila* and members of the* M. avium* complex, are believed to increase their own virulence during passage through their amoebal hosts [19, 20]. FLA, therefore, can act as proliferators and distributors of pathogenic bacteria in water systems other than being pathogenic themselves.

International research programs have consequently focused on the coexistence of FLA and ARB and the effects this relationship might have on traditional water quality testing techniques which look for the presence/absence of faecal indicators and protozoan parasites [15, 19]. Amoebal enrichment techniques have been used successfully, to selectively grow FLA and recover ARB from environmental samples [15, 21]. However, no studies to date in South Africa have applied amoebal enrichment techniques to selectively grow indigenous FLA in water systems, presenting a need to optimize this technique using local conditions.

Although the presence of FLA in natural environmental waters and manmade water systems has been well documented worldwide, few studies have reported on the occurrence of FLA in wastewater treatment plants [22–24]. Therefore, there is a need to obtain more information regarding the occurrence of FLA in wastewater treatment plants. This work included in this study is the first to determine the occurrence of FLA in a wastewater treatment plant in South Africa. The investigations in this study are divided into two parts: the first includes the optimization and establishment of amoebal enrichment techniques to isolate FLA under laboratory conditions using seeded samples, whereas the second includes the application of optimized conditions to isolate FLA potentially containing pathogenic ARB, at different stages of a wastewater treatment plant taking into consideration seasonal differences.

## 2. Materials and Methods

### 2.1. Optimization and Seeding Experiments

This study was an exploratory study to investigate the possibility for the presence of amoebae. It was decided to use* A. castellanii* as it is easily identified morphologically (the basis of isolation in this study) compared to other FLA which require further methods like polymerase chain reaction (PCR) to confirm them. The most appropriate temperature, food source,and concentration method for growth of* Acanthamoeba castellanii *(ATCC 30010) type strain were determined as indicated below.

#### 2.1.1. Optimization of Laboratory Conditions for* A. castellanii* Growth

The type strain* Acanthamoeba castellanii* Neff (ATCC 30010) was obtained from the American Type Culture Collection (Rockville, MD, USA). The strain was reconstituted and grown in tissue culture flask (Nunc, USA) containing 5 mL plate count broth (PCB) (Merck, SA) according to the manufacturer's instructions. The reconstituted amoebae were incubated in triplicate experiments at three temperatures including room temperature, 32°C, and 37°C. The growth medium was replaced at weekly intervals to maintain a constant supply of fresh axenic amoebal trophozoites. In order to compare membrane filtration and centrifugation as methods for sample concentration, two split samples were prepared from 500 mL water samples which were seeded with* A. castellanii* (ATCC 30010). One portion was concentrated by membrane filtration through 0.45 *μ*m pore size cellulose nitrate membranes (Millipore, SA) and the other by centrifugation at 1000 g for 20 minutes (Biovac Neofuge 15R, Vacutec, SA). Membrane filtration and incubation at 32°C were found to be optimal and thus used for further experiments. In order to determine the most appropriate food source for recovering amoebae, the concentrated samples were inoculated into living or heat-killed* E. coli.* The* Escherichia coli* (ATCC 25922) type strain was obtained from the American Type Culture Collection (Rockville, MD, USA). The type strain was reconstituted and maintained according to supplier's instructions before being inoculated onto nutrient agar (NA) and incubated at 37°C overnight. The stock plates were sealed and stored at 4–10°C until use. The type strain was maintained by weekly subculturing onto fresh NA plates. Heat-killed* E. coli *was prepared by placing a suspension of the type strain (*E. coli*) in a boiling water bath for 20 minutes immediately before use. Non-nutrient agar (NNA) plates were inoculated with 100 *μ*L of living or heat-killed E. coli (HKEC) by spreading the suspension evenly over the surface. For quality control purposes, nutrient agar plates were also inoculated with HKEC and incubated overnight at 37°C.

One split sample (500 mL) was passed through a cellulose nitrate membrane (Millipore, SA) with a pore size of 0.45 *µ*m. The membrane was cut into three pieces and each piece was placed upside down onto a NNA-*E. coli* plate. The plates were incubated aerobically at room temperature (22–25°C), 32°C, and 37°C. The other split sample was centrifuged at 1000 g for 20 minutes as recommended in the Health Protection Agency protocol [25]. The supernatant was aseptically removed by aspiration leaving approximately 2 mL covering the pellet. This was mixed thoroughly; 100 *µ*l of this mixture was inoculated onto NNA-*E. coli* plates and incubated aerobically at 32°C, 37°C, and room temperature.

#### 2.1.2. Seeding Experiments

The optimized food source and temperature were used for the growth of* A. castellanii *as mentioned above. Trophozoites were harvested by centrifugation at 1000 g for 20 minutes to obtain a pellet. The pellet was washed three timeswith 1 mL sterile Page's amoebal saline (PAS) and centrifuged at 1000 g for 20 minutes after each wash. The resulting pellet was resuspended in 1 mL sterile PAS. Nine sterile distilled water samples (500 mL each) were then seeded with 100 *µ*L suspension of* A. castellanii. *After seeding, each water sample wasdivided into 10 equal portions of 50 mL to represent split samples (*n* = 90); each seeded sample was treated identically thereafter.

### 2.2. Amoebal Enrichment

Amoebal enrichment technique used was adapted from previous studies [2, 21]. Briefly, the 90 from seeding experiments were concentrated by filtration using a 0.45 *µ*m pore size cellulose nitrate membrane (Millipore, SA). The membrane was placed upside down onto a NNA-HKEC plate with a few drops of sterile PAS, incubated aerobically at 32°C, and checked daily under light or inverted microscope for the appearance of amoebal trophozoites and cysts. The density of amoebal growth on the plates was recorded as (the average in 10 fields) <10 per field (+), 10–100 per field (++), or >100 per field (+++). Plates with amoebal growth were purified by aseptically cutting small agar plugs, placing them upside down onto fresh NNA-HKEC plates, and incubating as before. Once purified, amoeba were removed from the agar by gentle scraping, resuspended in sterile PAS, and washed at least three times at 1000 g for 20 minutes to remove extracellular bacteria and debris. The concentrate was then resuspended in 1 mL sterile PAS, inoculated into a sterile 24-well flat-bottomed microtiter plate (Nunc, USA), and again incubated at 32°C. The plates were checked for the morphological appearance of trophozoites and/or cysts under an inverted microscope (Leica, Germany), equipped with a 40x objective, at regular intervals. Fifty microliters of the amoebae suspension was harvested from the microtiter plate, heat-fixed on microscope slides, and Giemsa-stained to screen for the presence of amoebal trophozoites and/or cysts.

### 2.3. Environmental Samples

#### 2.3.1. Sample Collection

**Figure 1:**
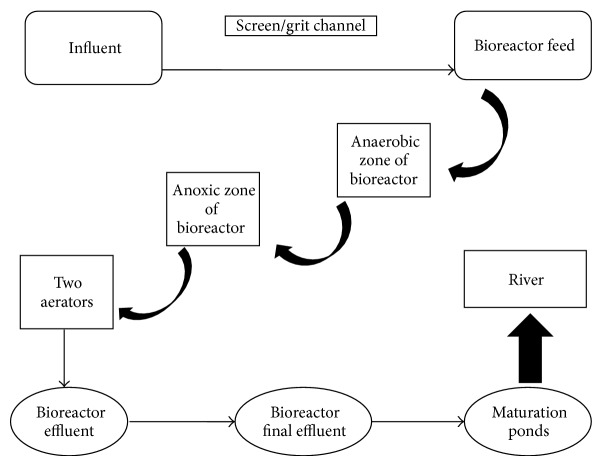
Schematic diagram of wastewater treatment plant indicating the sampling points of the present study.

The wastewater treatment plant (Figure 1) consists of a screen/grit channel, primary sedimentation, thickeners for raw sludge, thickeners for waste activated sludge, and bioreactors incorporating the three stages, configuration, final clarification, and maturation ponds. A total of 172 samples were collected over 4 seasons: autumn (41), winter (43), spring (44), and summer (44) during May, July, September, and November of 2010 at a wastewater treatment plant in Gauteng, South Africa. The samples were collected over different days for a total in a month/season. Along the treatment plant, samples (500 mL each) were collected from influent (16), bioreactor feed (20), anaerobic zone of the bioreactor (16), anoxic zone of the bioreactor (16), the two aerators (32), bioreactor effluent (16), bioreactor final effluent (11), and the maturation ponds (45).

The concentrations of chlorine residual in the treated effluents were determined on-site using the Lovibond Comparator system 2000 (Cydna laboratory, SA). Sample bottles for final effluent and maturation ponds contained 0.1% sodium thiosulphate (3% solution) to neutralize residual chlorine. At each sampling point, the temperature and pH were recorded on-site, respectively, with a portable thermometer and pH meter. Samples were processed within 24 hours of collection.

#### 2.3.2. Sample Processing

All samples were analyzed according to the methods established with seeded samples. Samples (500 mL each) were filtered through a 0.45 *µ*m pore size cellulose nitrate membrane (Millipore, SA). The membrane filters were then inoculated onto NNA-HKEC plates and incubated at 32°C to allow for the growth of indigenous amoeba. When amoebal trophozoites and/or cysts were observed, they were subcultured by aseptically cutting small agar plugs, placing them upside down onto fresh NNA-HKEC plates. Subculturing was repeated 3 to 4 times to allow purification of amoebae isolates. Once purified, amoebae were resuspended in sterile PAS, inoculated into a sterile 24-well flat-bottomed microtiter plate (Nunc, USA), and again incubated at 32°C. The plates were checked daily for the morphological appearance of trophozoites and/or cysts under an inverted microscope (Leica, Germany), equipped with a 40x objective. A suspension of 50 *µ*L from the microtiter plate containing the amoebae was heat-fixed on microscope slides and Giemsa-stained to screen for the presence of amoebal trophozoites and/or cysts potentially containing intracellular bacteria.

### 2.4. Molecular Methods

#### 2.4.1. DNA Extraction

A total of 30 environmental samples, 22 positive for* Acanthamoeba* sp. and 8 random samples negative for* Acanthamoeba* sp., were selected for molecular analysis. Amoebae DNA were extracted without pretreatment from environmental samples. Volumes of 700 *µ*L of the sample were centrifuged for 2 minutes at 12000 ×g to concentrate cells. The supernatant was discarded and 700 *µ*L of the lysis buffer was added to the pellet, mixed, and incubated for 10 minutes at 70°C. Volumes of 250 *µ*L of 100% ethanol were added and incubated at 56°C for another 10 minutes. To prepare the spin column, 50 *µ*L of celite was added, vortexed, and incubated at room temperature for 30 minutes with mixing every 30 s. The spin column was then placed in a clean 2 mL Eppendorf tube. A third of the solution (400 *µ*L) was loaded into the spin column, centrifuged for 30 s at 12000 ×g before discarding the elute (this step was repeated until the column was fully loaded). Wash buffer (400 *µ*l) was added, centrifuged for 30 s at 12000 ×g before discarding the elute (this step was repeated). Volumes of 400 *µ*L of 70% (v/v) ethanol were then added to the column, centrifuged for 30 s at 12000 ×g before the elute was discarded (this step was also repeated). The column was dried by centrifuging at 12000 ×g for 2 minutes before being transferred to a clean 1.5 mL Eppendorf tube. TE (Tris and EDTA) buffer (100 *µ*l) was then added to the column and incubated for approximately 2 minutes at 56°C. The column was discarded after the solution was centrifuged for 2 minutes 12000 ×g. The extracted DNA was then stored at −20°C and used for further applications. DNA was quantified using the Quanit-It HS assay kit (Invitrogen, SA) according to the manufacturer's instructions.

#### 2.4.2. PCR Assays

Polymerase chain reactions were performed in 50 *µ*L reaction tubes containing specific primer sets as described elsewhere [2]. The primer set, Ami6F1 5′CCAGCTCCAATAGCGTATATT3′ and Ami9R1 5′GTTGAGTCGAATTAAGCCGC3′, was used to amplify the 18S rRNA gene. These primers yield a fragment of approximately 700 bp. The primers at a concentration of 10 *µ*M each were transferred into a reaction tube containing 10 *µ*L template DNA, 2 mM MgCl_2 _, 2.5 U of taq DNA polymerase (Life Technologies, SA), 100 *µ*M each deoxynucleoside triphosphate and 8 *µ*L ultrapure water (Fermentas, Canada), and 0.2 *µ*L hotStar Taq Polymerase. A positive control was used in each experiment which comprised all the reagents mentioned above other than template DNA which was replaced with 10 *µ*L of genomic DNA extracted from the reference strains of* Acanthamoeba* spp. A negative control was also used in each experiment which comprised all the reagents mentioned above other than template DNA which was replaced with 10 *µ*L of PCR water. To detect* Acanthamoeba* sp., the reaction tubes were initially activated at 95°C for 15 minutes followed by 40 cycles of amplification using denaturation at 94°C for 45 s. Annealing was done at 57°C for 45 s and extension at 72°C for 1 minute followed by a final extension cycle at 72°C for 3 minutes. DNA was analyzed in a horizontal 1% (w/v) agarose slab gel (FP Agarose from Promega) with ethidium bromide (0.5 *µ*g/mL) in a TAE (40 mM Tris acetate; 2 mM EDTA, pH 8.3) buffered system. 5 *µ*L of 100 bp DNA marker (Fermentas O'GeneRuler DNA ladder, Canada) was loaded into the first well of the gel and into the remaining wells 10 *µ*L each sample (including positive and negative controls) mixed with 3 *µ*L of loading dye (Fermentas Orange × 6 Loading Dye, Canada).

### 2.5. Statistical Analysis

Statistical analysis was carried out to compare amoebae recovered using live or heat-killed* E. coli* at different temperatures (room temperature, 32°C, and 37°C) and concentration techniques (centrifugation or filtration). The Stata v11 statistical software was used and results were presented in a tabular format (STATA software, version 7.0; Stata Corporation, College Station, TX). Pearson chi-square test was used to test for association between categorical variables. The interpretation was performed at 95% confidence limit. All tests of significance and correlations were considered statistically significant at *P* values of <0.001.

## 3. Results 

### 3.1. Seeded Samples

**Figure 2:**
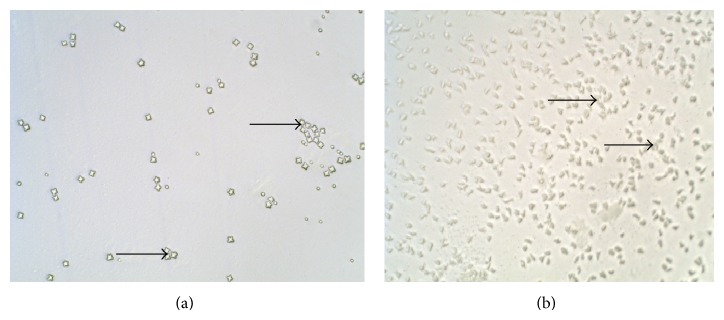
(a) Typical star shaped* Acanthamoeba* cysts (arrow) and (b)* Acanthamoeba* trophozoites (arrow) observed on HK-*E. coli*-NNA plates from all samples seeded with* A. castellanii* type strain, microscope at ×10 magnification (Leica, Germany).

*Acanthamoeba* was identified by both the polygonal shaped walls in the cyst form and the finger-like acanthapodia in the trophozoite form in all seeded samples (Figure 2). However, there were differences in the densities of amoebae recovered when using different concentration methods and incubation temperatures (Table 1). High densities of amoebae were observed when filtration was used as a concentration method, with only 2 plates classified as low, 25 as high, and 23 as very high. When centrifugation was used low densities of amoebae were observed with as many as 31 plates classified as low, 19 as high, and none as very high. Greater amoebae recovery densities were observed in samples incubated at 32°C and 37°C compared to those at room temperature (*P* < 0.001). There was, however, no significant difference between 32°C and 37°C with respect to amoebae densities, despite the fact that amoebae at 37°C encysted rapidly after 3 days compared to amoebae at 32°C which took one week to form cysts. No significant difference between live or heat-killed* E. coli* with respect to amoebae recovered was also observed. Consequently, we decided to use HK-*E. coli* for the following reasons: (i) to ensure that we do not observe actual “food” bacteria in the amoeba while setting up the method and (ii) also due to the high level of contamination in the wastewater samples, we felt that using HK-*E. coli* would be more appropriate to give a more reliable indication of intracellular bacteria in the samples.

### 3.2. Environmental Samples

#### 3.2.1. Physicochemical Parameters

The mean water temperature of samples taken at the wastewater treatment plant was 18.3°C, 12.5°C, 20.6°C, and 25.3°C in autumn, winter, spring, and summer, respectively (Table 2). The water temperature was significantly different (*P* < 0.0001) amongst the seasons when samples were collected, ranging from as low as 6.6°C in winter to as high as 27.7°C in summer. In contrast, pH was not seasonally dependent as the mean pH for autumn, winter, spring, and summer was 7.19, 7.28, 7.20, and 7.18, respectively. However, among the different sampling points the pH varied from 6.33 to 8.13 (Table 2).

#### 3.2.2. Concentration of Residual Chlorine in The Environmental Samples

Table 3 illustrates free chlorine residual concentrations at the different sampling points during the study period. Residual chlorine concentration ranged between 0.01 and 1.10 mg/L throughout the sampling period, with the final effluent having the highest concentration of 1.10 mg/L. The ranges for pond 1, pond 2, and river were below 0.1 mg/L. The mean chlorine residual concentration decreased from final effluent (0.37 mg/L) to maturation pond 3 (0.03 mg/L) where the treated water enters the river.

#### 3.2.3. Isolation of Free-Living Amoebae

**Figure 3:**
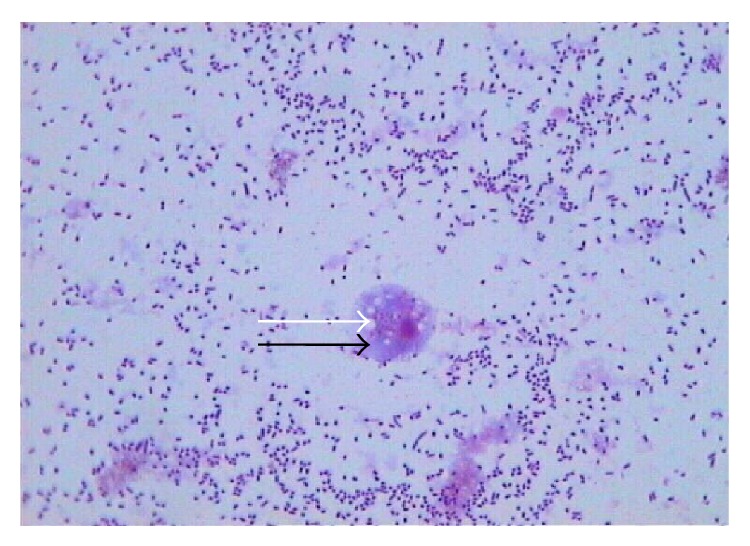
Amoebal trophozoite (black arrow) on Giemsa stain with round vacuoles (white arrow), ×100 (Olympus, Japan).

**Figure 4:**
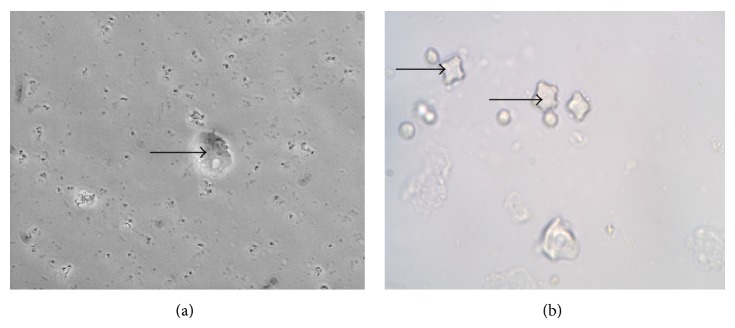
(a) Typical* Acanthamoeba* trophozoites (arrow) and (b) Typical star shaped* Acanthamoeba* cysts (arrow) observed on HK-*E. coli*-NNA plates from environmental samples, phase contrast ×40 (Leica, Germany).

Free-living amoebae identified on Giemsa stain were surrounded by numerous extracellular bacteria (Figure 3).* Acanthamoeba* sp. was identified by both the polygonal shaped walls in the cyst form and the finger-like acanthapodia in the trophozoite form in all the wastewater samples (Figure 4). A total of 150 (87.2%) samples were positive for free-living amoebae. Twenty-two (12.8%) samples were identified as* Acanthamoeba* sp. with the highest number recorded in autumn. Although FLA were isolated in 43 (100%) of the winter and spring samples, none of the samples had* Acanthamoeba* sp. which were only isolated in autumn, 21 (51.2%), and summer, 1 (2.2%) (Table 4).

**Figure 5:**
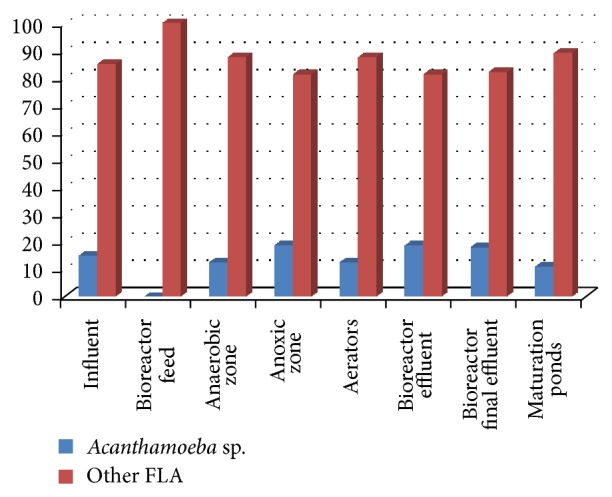
Percent of* Acanthamoeba* sp. and FLA isolated at different sampling points of a wastewater treatment plant in South Africa.

FLA were isolated in all sampled stages of the wastewater treatment plant using the amoebal enrichment technique. From Figure 5, a total of 16 (9.3%) samples collected were positive for FLA from the influent, 20 (11.6%) from the bioreactor feed, 16 (9.3%) from the anaerobic zone, 16 (9.3%) from the anoxic zone, 32 (18.6%) from the aerators, 16 (9.3%) from the bioreactor effluent, 11 (6.4%) from the bioreactor final effluent, and 45 (26.2%) from the maturation pond. No* Acanthamoeba* sp. was isolated in the bioreactor feed (Figure 5). Intracellular small bacterial-like organisms were observed in amoebae isolated in 30 (17.4%) of the environmental samples. According to the samples that were positive for intracellular bacteria, 24 (80%) were from autumn with the rest of samples spreading evenly among the other seasons.

#### 3.2.4. Detection Of FLA by PCR

**Figure 6:**
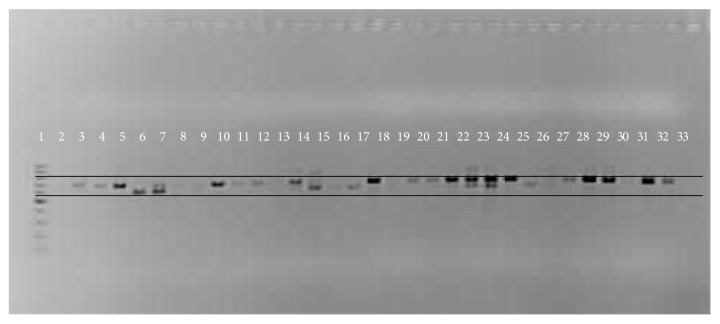
Gel picture showing PCR amplification of* Acanthamoeba* using primers Ami6F1 and Ami9R1, 1: 100 bp ladder (Fermentas), 2: negative control, and 3: positive control.

All samples detected morphologically as* Acanthamoeba* sp. were also positive using PCR. Only one of the eight samples not detected by morphology as* Acanthamoeba* sp. was positive using PCR. The primer set, Ami6F1 and Ami9R1, amplified a 700 bp (approximate) fragment for* Acanthamoeba* DNA. The positive control (*A. castellanii*) was amplified while the negative control (distilled water) was not amplified (Figure 6).

## 4. Discussion

### 4.1. Optimization of Conditions for Amoebal Enrichment

The amoebal enrichment technique has been used to isolate FLA from environment using different conditions for concentration, temperature, and food source. Well-known concentration methods, membrane filtration and centrifugation, have been used to isolate and concentrate FLA in environmental water samples. In the present study, the efficiency of these concentration methods in recovering amoebae in seeded water samples was compared. Results showed that samples that were filtered significantly recovered more amoebae as compared to samples that used centrifugation as a concentration method. Our findings are similar to those reported by the Health Protection Agency [25] in which membrane filtration was found to be more efficient in recovering amoebae when compared to centrifugation. A study by [26] has also shown that amoebae can be recovered more easily when samples are processed by filtration rather than centrifugation. However, the concentration method used also depends to some extent on sample volumes. Studies that have used centrifugation have concentrated relatively small sample volumes of 50 mL compared to filtration which has been used to concentrate relatively high sample volumes of 500 mL and 1000 mL [23, 27]. Volumes of 500 mL used for concentration in our study might explain why filtration was more efficient in recovering amoebae compared to centrifugation. The amoebae yield by centrifugation could therefore be improved by dividing the sample into smaller sample portions. There is no consensus in studies required to obtain a high recovery of amoebae on the speed and the time used when centrifugation is used. A report by the Health Protection Agency [25] recommends that water samples be centrifuged at 750 g for 20 minutes. However, studies by [28, 29] have used 120 g for 10 minutes and 1200 g for 15 minutes, respectively, to concentrate amoebae from environmental samples.

Temperature is another important factor that influences the growth of amoebae. For example, a study by Khan [30] has showed that nine strains of* Acanthamoeba* sp. grew at temperatures ranging from 10 to 37°C, with the pathogenic varieties surviving at higher temperatures (>37°C). This study indicates a relatively wide temperature range tolerated by amoebae. Environmental* Acanthamoeba* isolates of a study in Slovakia have also been shown to grow at 23°C, 32°C, and 37°C [31]. These findings agree with our study in which growth of amoebae was established at room temperature (22°C–25°C), 32°C, and 37°C. However we managed to recover more amoebae at 32°C and 37°C compared to room temperature which had a low recovery of amoebae. Although* A. castellanii* formed cysts after 3 days of incubation at 37°C, its ability to grow at this temperature showed its pathogenic potential for humans. With these findings, this study showed that amoebae can be grown at 32°C and/or 37°C giving important indications of the pathogenic potential of these organisms.

In general, FLA and* Acanthamoeba *sp. can be readily cultivated on nonnutrient agarcontaining a lawn of killed or living Gram negative bacteria. In the present study, heat-killed (HK)* E. coli* and living* E. coli* were compared as food sources for amoebal growth. There was no significant difference in the amoebaerecovered when alive or HK-*E. coli* were used as food sources. In contrast, a study done by Pickup et al. [32] has shown growth rates of* A. castellanii* to be higher on living cells of* E. coli*,* P. aeruginosa*,* K. aerogenes,* and* S. aureus* compared with those on the heat-killed bacterial cells. The effectiveness of live and heat-killed bacterial suspensions on the growth of* Acanthamoeba* has also been shown in another study by Selvam et al. [33]. In their study, live* P. aeruginosa, E. coli, *and* Bacillus* sp. yielded a higher total number of* Acanthamoeba *compared to heat-killed* P. aeruginosa, E. coli, *and* Bacillus* species.

### 4.2. FLA in Wastewater

The occurrence of FLA such as* Acanthamoeba *and* Naegleria* species has been previously reported in manmade sources like cooling towers, swimming pools, hospital water networks, and drinking water plants as well as in natural sources like rivers and lakes. However, few studies have reported on the occurrence of FLA in wastewater treatment plants and particularly in sewage water. Bose et al. [22] did a characterization study of potentially pathogenic FLA in sewage samples which resulted in the isolation of a pathogenic strain of* A. castellanii* and a nonpathogenic strain of* A. astronyxis*. Another study done by Ramirez et al. [23] isolated thirteen species of FLA (pathogenic and nonpathogenic) that included three species of* Acanthamoeba: A. castellanii, A. culbertsoni, *and* A. polyphaga, *from an activated sludge plant. A more recent study by García et al. [24] characterized potentially pathogenic* Acanthamoeba*,* Hartmannella,* and* Naegleria *from sewage effluents of Spanish wastewater treatment plants despite disinfection with chlorine. In the present study, FLA and* Acanthamoeba* sp. were isolated at different stages of a wastewater treatment plant (including sewage effluents), showing consistence with previous studies. Using the culture-based method, amoebal enrichment, this study focused on* Acanthamoeba* sp. which was further confirmed by PCR. It is well known that PCR methods are more rapid and capable of detecting even nonculturable cells and allow genus discrimination of the isolates [34]. The sensitivity of the molecular analysis is supported in our study by one positive sample for* Acanthamoeba* sp. out of the eight analyzed which could not be detected by amoebal enrichment. However, amoebal enrichment makes the organism available for further classification and allows testing of infectivity in human macrophages and testing antibiotic susceptibility [15].

All samples collected in the present study were positive for FLA in all the four seasons. However,* Acanthamoeba* sp. was only isolated during the autumn and summer in our study compared to a study by Ettinger et al. [35] which isolated* Acanthamoeba* sp. in spring and summer. This shows that seasonal changes may affect the prevalence of FLA in environmental water as the temperature fluctuates. Isolation of amoebae from chlorinated samples in our study shows that some amoebal cells can survive the wastewater treatment process even after chlorination, resulting in the discharge of amoebae to receiving water bodies such as rivers. The survival of amoebae to chlorination is because of amoebal cyst walls containing cellulose that forms a physical barrier against chlorine [18]. The chlorine residual concentration in this study ranged between 0.01 and 1.10 mg/L in the final effluent which fell well outside the limit of free chlorine residual applicable in South Africa. The residual chlorine range of discharged wastewater into a water resource such as a river should vary from 0.3 mg/L to 0.6 mg/L [36]. However, a study done by Storey et al. [11] showed that amoebal cysts can survive chlorine concentrations as high as 100 mg/L for 10 minutes. Therefore, the currently applied effluent concentrations of free chlorine residual may not result in the inactivation of amoebal cysts. In addition, Thomas et al. [12] also demonstrated that FLA, including* Acanthamoeba* sp.,* Hartmannella* sp., and* Vahlkampfia* sp., can resist treatment with ozone, chlorine dioxide, monochloramine, copper-silver, and chlorine.

The resistance of FLA to biocidal treatments such as chlorine has major implications for disease transmission as some FLA species can potential infections of the central nervous system, skin and eye. In addition to their role as pathogens, FLA are known to serve as natural hosts and vectors of various pathogenic intracellular bacteria [15]. In the present study, typical amoebal trophozoites and cysts containing live bacteria were observed in 30 (17.4%) of the environmental samples suggesting the intracellular existence of these amoeba resistant bacteria. The number of samples positive for intracellular bacteria in this study might also be underestimated because of the presence of other organisms in the environmental samples which may have skewed our results. However, what is important is that even if the number of positive samples was underestimated, 17.4% were still a relatively high number of samples to contain potential pathogenic intracellular bacteria. This in turn reduces the microbiological quality of the receiving water body (river in this case) as pathogenic microorganisms are released from the water treatment plant. This also increases the risk to the health of communities living in the vicinity of the river that uses the water for multiple purposes which include drinking, agricultural, and recreational purposes [37].

## 5. Conclusion

In this investigation, the amoebal enrichment technique was successfully optimized with seeded samples using filtration as a concentrating method, HK-*E*.* coli* as a food source for amoebae, and 32°C and/or 37°C as the incubation temperature. Using these optimized amoebal enrichment conditions, FLA (with some harboring potential pathogenic bacteria) were detected at all stages of the wastewater treatment plant.* Acanthamoeba *sp. was only detected in summer and autumn, showing that their prevalence is temperature dependent. The presence of amoebae in 87.2% of the environmental samples in this study shows that the current wastewater treatment process is not adequate for the removal and disinfection of amoebae using chlorine. Future studies should focus on the detection of FLA in wastewater effluents using both culture and molecular analysis to identify potentially pathogenic FLA entering receiving water bodies where humans could be exposed.

## Figures and Tables

**Table 1:** Densities of amoebae recovered using different food source, concentration methods and temperatures.

Condition	Amoebae recovered (no. of plates)		
	Low (+)	High (++)	Very High (+++)
Live *E. coli *	0	29	21
HK *E. coli *	1	29	20
Filtration	2	25	23
Centrifugation	31	19	0
RT	15	24	11
32°C	0	28	22
37°C	0	32	18

RT = room temperature.

**Table 2:** Water temperature and pH during sampling for different seasons.

Sampling season	Sample number	Water temperature (°C)		Water pH	
		Range	Mean	Range	Mean
Autumn	41	12.6–22.9	18.3^a^	6.33–7.90	7.19
Winter	43	6.6–18.2	12.5^b^	6.65–8.13	7.28
Spring	44	12.7–23.6	20.6^c^	6.67–7.76	7.20
Summer	44	21.8–27.7	25.3^d^	6.77–7.70	7.18
Probability (*P*)			<0.001^e^		NS

a, b, c, and d: mean values in the same column not sharing the same superscript are statistically significantly different *P* < 0.0001.

e: *P* values of <0.001.

NS: not statistically significant difference.

**Table 3:** Concentrations of free residual chlorine at different sampling points.

Sample source	Free chlorine residual (mg/L)	
	Ranges	Means
Effluent	0.05–1.10	0.37
Maturation pond 1	0.02–0.07	0.04
Maturation pond 2	0.01–0.05	0.03
Maturation pond 3	0.01–0.05	0.03

**Table 4:** Isolation of amoebae at different seasons in a wastewater treatment plant.

Season	Sample number	*Acanthamoeba* sp.	Other FLA
Autumn	41	21 (51.2%)	20 (48.8%)
Winter	43	—	43 (100%)
Spring	44	—	44 (100%)
Summer	44	1 (2.2%)	43 (97.8%)
